# From Valuation to Governance: Using Choice Experiment to Value Street Trees

**DOI:** 10.1007/s13280-014-0516-9

**Published:** 2014-04-17

**Authors:** Marek Giergiczny, Jakub Kronenberg

**Affiliations:** 1 Faculty of Economic Sciences, University of Warsaw, Dluga 44/50, 00-241 Warsaw, Poland; 2 Faculty of Economics and Sociology, University of Lodz, POW 3/5, 90-255 Lodz, Poland

**Keywords:** Urban ecosystem services, Choice experiment, Street trees, Poland

## Abstract

This paper reports a choice experiment used to estimate the value of street trees in the city center of Lodz, Poland, and the broader context of how valuation results helped to improve governance of urban ecosystem services in this city. Based on a simplified inventory of trees, we prepared a set of hypothetical programs which put varying emphasis on the different ways to increase the numbers of trees, along with different levels of a hypothetical tax that would have to be paid by respondents to implement a given program. Our study indicated that the 351 surveyed Lodz residents were willing to pay the highest price for greening those streets where currently there are few or no trees and confirmed the general importance of planting trees. The results provided an argument in the debate on the new development strategy for the city and helped to promote the concept of ecosystem services.

**Electronic supplementary material:**

The online version of this article (doi:10.1007/s13280-014-0516-9) contains supplementary material, which is available to authorized users.

## Introduction

While globally there is a growing interest in the importance of green infrastructure for the quality of life in cities (Hubacek and Kronenberg 2013), in some regions, such as Central and Eastern Europe, urban green areas are shrinking, despite the physical growth of cities (Kabisch and Haase 2013). Street trees are often presented as an easy to understand and well-known source of benefits (McPherson 2007, 2010), but they are also particularly exposed to the risks of urbanization (e.g., pollution and modernizing or developing infrastructure). People tend to protest when street trees are removed, including in Central and Eastern Europe, but they do so on a case by case basis, and usually without reference to the broader scale of this problem. In this light, it is interesting to study more systematically if street trees are important to people in cities where urban green areas are shrinking. In other words, it is useful to know if the trend of shrinking urban green areas is in line with people’s preferences, or if it is something that people would prefer to counteract.

Street trees are specific in that while most of them are public, some of their services and disservices accrue only to those who live nearby (Donovan and Butry 2010). Moreover, the perceptions of street trees in a city center might vary considerably between people living in the center and outside of it. Economic valuation methods can be used to study such various aspects of the management of urban trees, and some of them are particularly suited for this purpose.

The objectives of this article are to investigate the usefulness of one of these methods, choice experiment, to estimate the economic value of street trees in a city center, and to test this approach in the context of an understudied region of Central and Eastern Europe. Using a case study of Lodz in Poland, we investigate the specific implications of this valuation exercise for governance of urban ecosystem services. To the best of our knowledge, there have been few studies that looked at the value of trees in a city center and no study used a choice experiment to value street trees. Furthermore, we were only able to identify two studies on the value of urban ecosystem services in Central and Eastern Europe: Melichar and Kaprová (2013) performed a spatial hedonic analysis of urban green spaces in Prague, while Pavlyuk and Jankowska (2012) used a choice experiment to assess the value of different types of management of urban and suburban forests in Riga.

Before we move to a description of our study, its results and implications for governance in the following sections, we first compare the different methods used to value urban street trees.

### Methods Used to Value Street Trees

In North American cities, tree valuations have been mostly based on replacement cost or avoided cost methods, thus offering an opportunity to investigate the monetary value of individual trees. The authors of these studies differentiate between what they call benefit-based and cost-based approaches (McPherson 2007). “Cost-based approach” uses the trunk formula method developed by the Council of Landscape and Tree Appraisers for calculating the replacement cost of a tree. “Benefit-based approach” focuses on the net present value of a stream of benefits that a tree is expected to deliver (avoided costs of emissions of CO_2_ and other pollutants, of energy use, and of stormwater management; increased property value), less the costs of the tree’s maintenance. Thus framed benefits are easy to grasp by decision makers and in some instances they served to underpin important political commitments to increasing the numbers of trees, for example, in New York City (McPherson 2010). However, both approaches are entirely expert led and do not investigate the preferences of city inhabitants.

One of the most common methods to estimate the value of urban greenery based on revealed preferences of city inhabitants is hedonic pricing, the use of which in the context of urban trees dates back to the 1970s (Payne and Strom 1975; Morales et al. 1976). Very rarely, however, this method has been applied specifically to street trees (Donovan and Butry 2010; Pandit et al. 2013), and these studies focused on residential areas, not necessarily in city centers. For example, in twelve Japanese cities, increasing street greenery was found to have higher impacts on land prices, than increasing the amount of urban park area (Ishikawa and Fukushige 2012). Nevertheless, there may always be some additional factors which account for the price differences among the analyzed properties and which have not been included in a model (Ishikawa and Fukushige 2012). Furthermore, Orland et al. (1992) called for caution in linking property prices with the adjacent trees in hedonic pricing models, based on their empirical study on the perceived impacts of street trees on property values.

Qualitative methods were also used to perform non-monetary valuation of street trees, involving various survey procedures for assessing people’s response to street trees (Getz et al. 1982; Sommer et al. 1990; Todorova et al. 2004; Lohr et al. 2004; Schroeder et al. 2006). Some of these authors investigated general perceptions of street trees, others focused on specific issues. Most of qualitative studies confirm positive attitudes of urbanites toward trees in different geographical contexts. Qualitative, non-monetary studies depict aspects which are difficult to grasp with the use of monetary valuation methods. For example, people who volunteered in urban tree planting programs were found to be motivated more by values, such as spiritual benefits and bringing nature closer, than by practical benefits, such as reducing noise and increasing property value (Westphal 1993; Austin 2002). It was also found that the perception of street trees may depend on whether one has contact with these trees (Gorman 2004).

In a country such as Poland, where it is often suggested in public discussions that people cannot afford the “luxury” of environmental protection (or just do not want to “waste” their money on it), it is particularly interesting to investigate the actual preferences in monetary terms. Furthermore, not all urbanites buy houses or apartments and thus their preferences would not be depicted using hedonic pricing. Among the stated preferences valuation methods, choice experiments are considered to be able to elicit these preferences in the most complex and comprehensive manner, permitting to study preferences for different attributes of a good (Hanley et al. 1998).

Choice experiments were very rarely used in the case of urban green spaces and, to the best of our knowledge, so far they have not been applied specifically to street trees. Bullock (2008) investigated the Dubliners’ value ranking of different aspects of urban parks, such as size, type, opportunities for diverse activities, etc., and identified their willingness to pay for visiting parks of different characteristics as compared to a baseline park with only some of those attributes. Other applications of choice experiments in urban settings included studies on the public rights of way in the UK (Morris et al. 2009), multiuse trails in urban and suburban environments (Reichhart and Arnberger 2010), preferences for environmental amenity improvements related to regeneration initiatives (Lanz and Provins 2011), and urban stream restoration (Bae 2011). Scenarios put forward during choice experiments are often built based on previous work with experts or focus groups (Davies and Laing 2002).

Taking into consideration the above features of different methods used to estimate the value of urban trees, we decided to use a choice experiment. It seems to be an innovative approach to value street trees in a city center, and it provides meaningful information that can constitute an input to local decision making and support local governance of ecosystem services. We wanted to get to know how city inhabitants value street trees and to check whether the current approach of city authorities to street trees corresponds with those preferences.

## Materials and Methods

### Choice Experiment

Choice experiment surveys have been used for many years in transport economics and market research and are now becoming increasingly popular for the valuation of environmental and health-related goods. They present sampled respondents with different choice sets, each comprising a finite set of alternatives defined on a number of attribute dimensions, and require respondents to specify their preferred alternative in each choice situation. Each alternative involves a bid amount to be paid, which generally equals zero for the Status Quo (SQ), as well as the level of each relevant non-monetary attribute of the good. The respondent’s task is to state his/her preferred option (Hensher et al. 2005). This setting is consistent with the random utility maximization model and several econometric treatments have been developed to analyze data from choice experiments. More information on choice experiment and the specifications of our model is available as Electronic Supplementary Material.

### Study Area and Scenario Development

Lodz is the third largest city in Poland and it is the city with the smallest area of streetside greenery among major Polish cities. The numbers of urban trees in general and street trees in particular have been decreasing in Lodz (and in most Polish cities) in recent years and the living conditions for trees have worsened (Kronenberg 2012; Kabisch and Haase 2013). There is no inventory of street trees in the city, and management practices in the city center are largely restricted to removing trees that are judged “in bad condition” or because of new infrastructural developments. Compensation plantings are ordered only when healthy trees are removed and they mostly take place outside of the center.

Lodz, and especially its center, is widely perceived as gray and neglected, and it is suffering from unsatisfactory environmental health indicators. For example, in 2003 Lodz had the highest mortality rate due to respiratory diseases of both men and women among all Polish cities with more than 100 000 inhabitants (Wcisło 2008).

Our study focused on the city center within which the total length of streets is about 50 km. This is a densely built area. Many streets are lined with narrow strips of unpaved ground that used to be green, with lawns and trees, but from which the trees have been removed over time without ever being replaced. For the purposes of this study, we performed a rough analysis of streets in the city center, classifying them into four categories: (i) “High”—streets with many trees (10 or more trees per 100 m), currently 12 km; (ii) “Medium”—streets with medium number of trees (4–9 trees per 100 m), currently 10 km; (iii) “Islets”—streets with trees planted on islets, currently 0 km; and (iv) “No trees”—streets with no or few trees (0–3 trees per 100 m), currently 28 km.

After consultation with landscape specialists it turned out that, in the most optimistic scenario, the following improvements are possible in terms of planting trees: (a) upgrading a maximum of 8 km of streets from “Medium” to “High”; (b) upgrading a maximum of 20 km of streets from “No trees” to “Medium”; and (c) for 8 km of streets, it is not possible to plant enough trees to change their “No trees” status. The improvement from “No trees” to “Medium” can be achieved either by planting trees in the space between sidewalk and road (there is enough space for planting additional trees in this way along maximum 8 km of streets) or by creating islands in the parking places or on the road, which may be possible on maximum 12 km of streets. Table 1 presents the attributes and their levels used at the designing stage.

**Table 1:** Attributes and attribute levels used at the design stage

Attributes	Levels
Upgrade from *Medium* to *High*	+2 km
	+4 km
	+6 km
	+8 km
Upgrade from *No trees* to *Medium*	+2 km
	+4 km
	+6 km
	+8 km
Upgrade from *No trees* to *Islets*	+3 km
	+6 km
	+9 km
	+12 km
Monthly increase in local tax (*Cost*)	1.64 USD
	6.56 USD
	11.48 USD
	16.40 USD

The choice sets were generated following the Street et al. (2005) and Street and Burgess (2007) optimal-in-difference design approach. Each respondent was faced with 12 choice situations, involving the choice between the SQ alternative, with no tree planting program and no payment required, and three program alternatives. Respondents were asked to select the best alternative in each of 12 choice sets.

To make things simpler, in the questionnaire we translated the attributes and levels from design stage into following categories: (i) Length of streets with a high number of trees; (ii) Length of streets with a medium number of trees; (iii) Length of streets with islets; and (iv) Length of streets with no trees.

**Fig. 1:**
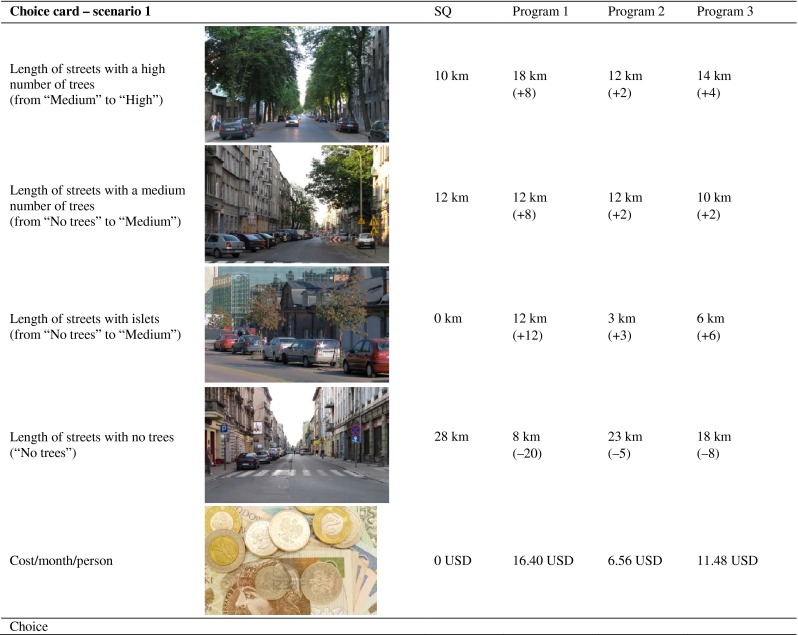
Sample choice card, with the original levels of attributes from the design stage shown in *brackets* (only total length of streets in each category was presented to respondents)

The payment vehicle used in the survey was monthly increase in the local tax that all Lodz citizens would have to pay.^1^ An example of a choice card is presented in Fig. 1.

The survey was conducted between July and November 2011. The questionnaire was administered face-to-face on a sample of the Lodz population, with interviews conducted in public places. Questionnaires were randomly assigned to 400 individuals and 351 valid questionnaires were collected and used in the econometric analysis described in this paper.

**Fig. 2:**
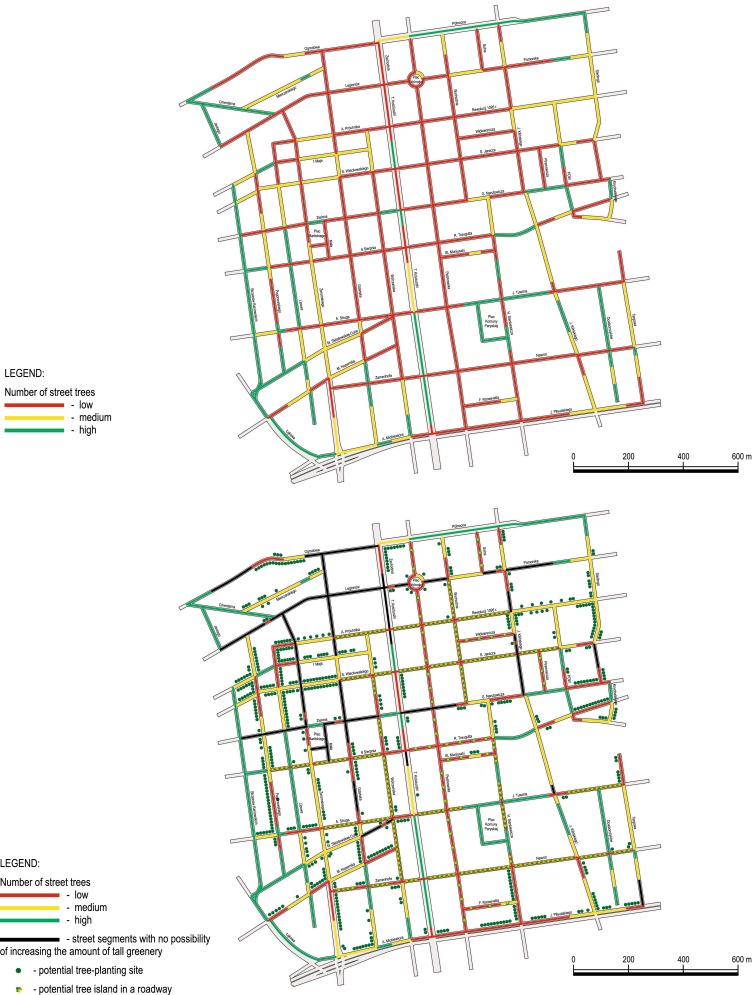
Maps of the city center of Lodz used with the questionnaires (the first map shows the city center and the *color* of each street indicated to which of our categories it belonged; the second map featured opportunities to upgrade each street using the different programs)

The questionnaire consisted of four parts. The first one included questions about respondent’s attitude toward trees in the city. The second part described the current situation in Lodz, using maps (Fig. 2) and photos to illustrate the attributes and their levels. The third part of the survey was the choice experiment. The forth part contained debriefing questions and collected socio-economic data, including gender, age, location, education, household characteristics, and income.

Two models were estimated on the data. We begin with a basic Multinomial Logit (MNL) model, with no random preference heterogeneity (model 1). This is then followed by a second model, which allows for random preference heterogeneity with correlation between individual coefficients, Mixed Multinomial Logit (MMNL).

The utility for the SQ alternative is given by a constant. The utility function for the three program alternatives includes continuous coefficients associated with:

*High*, upgrade from “Medium” to “High”;

*Medium*, upgrade from “No trees” to “Medium”;

*Islets*, upgrade from “No trees” to “Islets”;

and cost of the program.

In the MNL model, in addition to the main effects, we included four interactions of non-monetary attributes with the following socio-demographic variables: age, gender, education, and car ownership. By adding into the utility function the cost/income ratio we also allowed the cost sensitivity to vary with income level.

Finally, in all estimated models, we used a linear specification of attributes of the utility functions. This is based on preliminary analyses that did not reveal consistent and significant non-linearities in response with the data at hand. The modeling results are presented in Table 2. All models were coded and estimated in Nlogit 5.0.

**Table 2:** Modeling results showing that respondents would like to have more trees growing along the streets and that their preferences depend on the cost of a program, the respondents’ incomes, and socio-demographic characteristics (only in MNL model)

	MNL		MMNL	
	Coeff.	Asym. *t*-ratios	Coeff.	Asym. *t*-ratios
Main effects				
High	.2237**	6.64	.1273**	4.91
Medium	.1406**	5.20	.1046**	4.98
Islets	.0991**	6.02	.1192**	8.46
Cost	−.0167**	−8.55	−2.3965**	−22.29
SQ	−.4373**	−5.04	−1.3522**	−25.01
Socio-demographic effects				
Cost/income	−.0336**	−13.57	−.0138**	−4.46
Age*High	−.0059**	−12.23		
Age*Medium	−.0033**	−8.75		
Age*Islets	−.0015**	−6.32		
Edu*High	.0236**	2.85		
Edu*Medium	.0165*	2.41		
Edu*Islets	.0117**	2.92		
Car*High	−.01473	−1.07		
Car*Medium	−.01412	−1.00		
Car*Islets	−.0250**	−4.18		
Diagonal Cholesky				
High			.0362	1.29
Medium			.0532**	3.82
Islets			.0917**	7.25
Cost			.1872**	2.81
Below diagonal Cholesky				
MED:HIG			−.0373	−1.44
ISL:HIG			−.0778**	−4.00
ISL:MED			.0914**	8.52
COST:HIG			−3.3108**	−15.99
COST:MED			−1.5094**	−12.69
COST:ISL			−.4795**	−7.41
Standard deviations				
High			.03660	1.29
Medium			.06515**	3.20
Islets			.15022**	10.06
Cost			3.67517**	16.20
LL(β)	−5447.83		−3396.04	
Parameters	15		16	
Pseudo *ρ* ^2^(0)	.1471		.4692	
*N*	4212		4212	

** Significance at the 0.01 level, * significance at the 0.05 level

## Results

For both models, the signs of the main coefficients are the same and are consistent with a priori expectations. The estimate for the *SQ* constant is negative, indicating that respondents on average would like to move from the current situation to a program increasing the number of trees. The positive and statistically significant estimates for the fixed MNL coefficients and the MMNL means for *High*, *Medium*, and *Islets* imply that programs associated with larger upgrades to given street categories are more likely to be selected. The MNL model shows negative and significant cost sensitivity.

The signs and significance of interaction terms are consistent with a priori expectations. Respondents with a higher income have lower price sensitivity, this effect is statistically significant in both models. The interactions with socio-demographics are statistically significant only in MNL model so they were omitted in MMNL. Looking at MNL results, it can be seen that respondents with a higher education level (*Edu* was coded as years of education) have higher marginal utility associated with *High*, *Medium*, and *Islets*. Older respondents have ceteris paribus lower marginal utility associated with increasing the number of trees in all street categories. Respondents who own a car, have lower marginal utility associated with *High*, *Medium,* and *Islets*, interestingly this effect is statistically significant only for *Islets.*

In addition to the mean of the main effects, the MMNL model uses 10 extra parameters, these are the elements of the Cholesky matrix for the 3 normally distributed non-cost coefficients and for the log-normally distributed cost coefficient. We obtain a very large improvement in log likelihood by 2051.8 units when moving from MNL to MMNL, which is significant at high levels of confidence using a LR test.^2^ As shown in Table 2, a majority of the elements of the Cholesky matrix are significant at the 99 % confidence level, indicating that there is correlation among the random coefficients.

The standard deviations of the random parameters are statistically significantly different from zero at 99 % confidence for all parameters apart from *High*. The three normally distributed random coefficients have coefficients of variation (CV) ranging from 0.28 (*High*) to 1.26 (*Islets*). The highest CV for *Islets* is consistent with a priori expectations as creating islands was perceived by many respondents as the most controversial component of the program.

As a final step, we look at the WTPs calculated from the model estimates. The calculated trade-offs are reported in Table 3. Starting with the MNL estimates, it can be seen that, in both models, the mean WTP for all upgrades is positive and of similar magnitude. As expected, the highest heterogeneity in WTP is observed for *Islets*.

**Table 3:** Both models (MNL and MMNL) indicate that respondents were willing to pay (WTP in USD/month/km) for planting street trees, and the WTP values for all upgrades are very similar

WTP	MNL	MMNL		
	Mean	Mean	SD	Median
Upgrade to “High”	0.58	1.61	2.33	0.40
Upgrade to “Medium”	0.47	1.31	2.27	0.25
Upgrade to “Islets”	0.66	1.65	4.08	0.14

The relatively high CV for the WTP indicate that there is a substantial share of respondents who have negative estimates for the upgrades (i.e., 35 % for upgrade to “Islets” and 25 % for upgrade to “High”). These results are to a large extent consistent with the follow-up questions in which 18 % of respondents stated that the current number of trees in the city center is too large (21 % stated that it is sufficient and 61 % stated that currently there are too few trees).

The WTP values from MNL are presented as a reference level. Since MNL has some limitations, which are listed in the Electronic Supplementary Material, we focus mostly on the MMNL results. We only note that both models produce the same ranking of WTP values with WTP for upgrade to Islets being the highest and WTP for upgrade to Medium being the smallest.

The mean WTP values from the MMNL model are about 2.5 times larger than the values based on the MNL model. This is not surprising given that the cost is log-normally distributed.^3^ Hence the mean WTP values based on MMNL (which for all attributes are distributed as ratio of normal and log-normal distribution) can be to large extent influenced by a few very large WTP values. Having this in mind for the MMNL model, we also reported for each of the attributes the median WTP, which is a much more conservative measure.

## Discussion

Even though stated preference methods, including choice experiments, are perceived as particularly useful for valuing non-market goods and services, they are not free from problems. One of the general limitations of a stated preferences approach is its dependency on information quality and information interpretation by researchers and respondents. While we made every effort to ensure that information was clear and that the pollsters provided it in a consistent manner, there always remains a risk that some responses were “constructed in response to the information presented” (Burgess et al. 2000). Ultimately our results could be confirmed if a voting was performed on the question of introducing a tax that would fund planting trees in the city center (Schläpfer et al. 2004).

A particular advantage of using a stated preference method in Lodz was that it overcame problems with the availability of spatially explicit environmental data. Indeed, data availability is one of the crucial factors influencing the selection of a valuation method (Larson and Perrings 2013), and although a choice experiment is resource intensive, it is independent of previously collected data. Another advantage of choice experiments is that they avoid direct questions on the respondents’ WTP, which is often the case in standard contingent valuation and is more likely to lead to biased answers. Instead, respondents’ preferences are derived based on the trade-offs they make between different versions of a hypothetical scenario.

Our initial results were communicated to local stakeholders and were used to promote the concept of an economic value of urban ecosystem services in Poland. Although the numbers were perceived as impressive, they were not particularly surprising to most of the stakeholders. Trees are a powerful symbol and the benefits that they provide are relatively well understood, thus representing very well the concept of ecosystem services. Various studies indicated that people pay special attention to street trees in cities, and that streets in city centers rank very high as important places for government to provide trees (Getz et al. 1982). Nevertheless, for the purposes of enhancing governance in a setting where no other valuation study had been performed before, the general information provided by our survey results was sufficient to attract attention. The selection of a particular valuation method was probably not important from the perspective of those to whom we communicated our results because in most cases it was the first instance of valuation about which they had heard. In a case such as Lodz, from the perspective of enhancing governance, “some number is better than no number” as it serves to initiate discussion and raise interest.

By a lucky coincidence, the initial results became available at the time when the authorities of Lodz worked on an integrated development strategy for the city and a set of accompanying policies. Environmental issues were almost completely absent from the initial version of the strategy. Valuation results provided an important argument that could be used to promote environmental conservation and an alternative view on urban ecosystem services. They were used by the bottom-up movement to highlight the importance of ecosystem services in Lodz and the documents have been changed following public consultations within which these arguments have been voiced. Now, Lodz has relatively progressive documents which place the environment as one of the three pillars of development and explicitly refer to “the skillful use of ecosystem services and nature’s potential for sustainable development of Lodz as a compact city” (*Integrated Development Strategy of Lodz 2020*+). Although there is still a long way from strategy to implementation, this was an important step, together with putting the value of urban ecosystems on the agenda of local stakeholders involved in discussions on the development of Lodz. The relevant sectoral policy (*Municipal management and environmental protection policy of the City of Lodz 2020*+) starts with a reference to ecosystem services and focuses on ecosystem functioning and the benefits that we can obtain from ecosystems, if sound ecosystem management is ensured. Interestingly, in other cities in Poland, including those where linkages to ecosystems might seem much more obvious, such as coastal cities, ecosystem services have not been included explicitly in official documents (Piwowarczyk et al. 2013).

To translate the valuation results into implications for governance, local authorities should ensure that the residents’ opinions are listened to and that the residents’ involvement in planting trees is possible. Furthermore, especially in a situation where the budget is limited, city authorities should explore the possibility of creating opportunities for individuals or bottom-up organizations to plant trees at their own cost but in places arranged with the city. Although in Poland people may initially be reluctant to involve in such social activity because of the post-communist distrust to collective initiatives, experience from other countries suggests that planting trees at people’s own cost, in person or through participation in planting programs, enhances satisfaction with city trees and care for those trees afterward (Sommer et al. 1994; Summit and Sommer 1998). It may also increase the understanding of benefits provided by trees. Cities should create opportunities to make environmentally sound behaviors easier to engage in, or to make personal advantages resulting from such behaviors more clear to individuals (Summit and Sommer 1998). Indeed, many US cities which suffered from declines in tree numbers responded with large-scale tree planting initiatives (such as MillionTreesNYC or Million Trees Los Angeles), which rely to a significant extent on voluntary time and funding inputs from inhabitants.

Although both city authorities and other experts dealing with street trees in Polish cities perceive insufficient funding as the main barrier to harnessing the potential of urban trees to provide ecosystem services, they were not able to suggest where funds might come from apart from traditional local government resources or EU funds (Kronenberg 2012). Our study directly suggests that urban inhabitants would be willing to contribute to greening their cities (at least in Lodz), although this does not necessarily have to be through a tax but equally well through other types of initiatives within which city inhabitants could be involved by the authorities. In fact, in our study, the respondents might have had a natural incentive to reduce their declared WTP to avoid paying taxes. The fact that they still declared positive numbers indicates that they perceive the topic as highly important.

## Conclusion

Our study indicated the economic value of street trees to the inhabitants of Lodz, and that people would be willing to contribute financially to increasing the number of street trees in the city center. By a happy coincidence, the results were available in the important moment of discussion on a new city development strategy, the first version of which neglected environmental issues. Thus, these results gained publicity and complemented a discussion which helped to revise the strategy to better reflect the preferences of Lodz inhabitants. Indeed, the uptake of our results in Lodz confirms that the use of the concept of ecosystem services contributes to better understanding of the benefits of urban nature and reflects a general tendency to refer to the value of urban ecosystem services in various planning documents (Hubacek and Kronenberg 2013).

Further research would be useful on the value of street trees in Lodz, to compare our results with those of other valuation methods (hedonic, replacement cost, non-monetary). This would help to inform local decision makers and other stakeholders even better than the results of one method that we have used so far. Indeed, it is important to show this diversity of opportunities to decision makers and to inform them about the importance of valuation and the different options at their disposal, before they can start using these methods consciously as a basis for their decisions. It might also be useful to complement the current study with sociological research on why people express their preferences toward trees. This might help to adjust tree preservation strategies to the needs of different constituencies.

## Electronic supplementary material

Below is the link to the electronic supplementary material.

Supplementary material 1 (PDF 282 kb)

## Footnotes

1 Original values were expressed in PLN. USD values have been established using the average exchange rate from the data collection period (July–November 2011): 1 USD = 3.0496 PLN.

2 The improvement in Log likelihood is even larger (i.e., 2238) when the base model is the MNL model with main effects only, which is nested in MMNL.

3 See Giergiczny et al. (2012) for a detailed discussion of this topic.
