# Supplementary material for: From Valuation to Governance: Using Choice Experiment to Value Street Trees
Source: Ambio. 2014 Apr 17;43(4):492–501. doi: 10.1007/s13280-014-0516-9 (PMC3989514; doi:10.1007/s13280-014-0516-9)
Supplement: Supplementary file 1 — Supplementary material 1 (PDF 282 kb) [file 13280_2014_516_MOESM1_ESM.pdf]

AMBIO

Electronic Supplementary Material

From Valuation to Governance: Using Choice Experiment to Value Street Trees

Marek Giergiczny, Jakub Kronenberg

Choice experiment

In a choice experiment individuals are asked to identify their preferred choice i from a given set of J alternatives. The data analysis follows a RUM under which, it is assumed that the observed choice from individual n is the one he/she expects to provide him/her with the highest utility (McFadden 1974). His/her utility function, U_{ni}, can be decomposed into a systematic part, V_{ni} , and a stochastic part, ε_{ni}, i.e.: $U_{ni} = V_{ni} + \varepsilon_{ni}$.

The probability P_{ni} that individual n chooses alternative i instead of another alternative j of the choice set is: $P_{ni} = \Pr(V_{ni} + \varepsilon_{ni} > V_{nj} + \varepsilon_{nj} \forall j \neq i)$.

If ε_{nj} is assumed to be an independently and identically distributed extreme value type I, this probability has a closed form expression, $P_{ni} = \frac{e^{\beta' x_{ni}}}{\sum_j e^{\beta' x_{nj}}}$, where x is a vector of variables and β a vector of parameters. The above expression is often referred to as a logit choice probability function or Multinomial Logit Model (MNL).

The standard MNL has three limitations, as listed by Train (2003): (i) it exhibits a property of independence from irrelevant alternatives; (ii) MNL can represent only the systematic preference variation but not random preference variations; (iii) it cannot handle situations where the unobserved part of the utility function is correlated over time.

In addition to an MNL model, the data were analyzed with a mixed logit model, a model which relaxes the above limitations of MNL. Mixed logit probabilities can be expressed as the integrals of standard logit probabilities over a density of parameters. Following Train (2003), a multinomial mixed logit model (MMNL) is any model whose choice probabilities take the

form: $P_{ni} = \int \frac{e^{\beta_n' x_{ni}}}{\sum_j e^{\beta_n' x_{nj}}} \phi(\beta | b, \Omega) d\beta,$ where: $\frac{e^{\beta_n' x_{ni}}}{\sum_j e^{\beta_n' x_{nj}}}$ is a standard logit formula,

$\phi(\beta | b, \Omega)$ is the density of the random coefficients with mean b and covariance Ω.

In a standard MNL the unobserved factors that affect respondents are assumed to be independent over the repeated choices, which may be considered unrealistic as respondents usually make more than one choice. There might be some unobserved factors that are constant over the choices made by the same individual facing several choice sets, and consequently unobserved parts of the utilities over the choices may be correlated. Mixed logit models can account for dependence across repeated choices from the same respondent by specifying a panel version of the model. Conditional on β, the probability that the decision maker *n* makes a sequence of *T* choices is the product of logit formulas, $P_{ni} = \prod_{t=1}^{T} \left[\frac{e^{\beta_n' x_{nit}}}{\sum_j e^{\beta_n' x_{njt}}} \right]$,

where *t* denotes the sequence of choices made by the same respondent.

Since β_n is not known, the unconditional probability is given by the integral over all possible values of β_n, i.e. $P_{ni} = \int \prod_{t=1}^{T} \left[\frac{e^{\beta_n' x_{nit}}}{\sum_j e^{\beta_n' x_{njt}}} \right] \phi(\beta | b, \Omega) d\beta$, with $\phi(\beta | b, \Omega)$ being the density of a random parameter with mean *b* and covariance matrix Ω.

Distribution assumption in MMNL

There has been discussion in the field of choice modeling about acceptability of different distributions for the cost coefficient (e.g. Hensher and Greene 2003). As pointed by Daly, Hess, and Train (2012), this discussion has generally focused on the behavioral realism of

allowing for positive values in the distribution of the cost coefficient, rather than the possibility of non-existence of moments for the willingness to pay (WTP) distributions.

Daly et al. (2012) showed that some popular distributions used for the cost coefficient in random coefficient models, including normal, truncated normal, uniform and triangular, imply infinite moments for the distribution of WTP, even if truncated or bounded at zero. Daly et al. (2012) also presented a theorem that allows researchers to test whether the distribution of WTP has finite moments. Using this theorem, they showed that log-normal and Johnson's Sb distribution have all inverse moments in their basic specifications (i.e. with the domains between 0 and infinity for the log-normal, and 0 and 1, or any other positive number, for the Johnson Sb). Given that in the field of environmental valuation the moments of WTP distribution, especially the mean, are of crucial interest, we decided to assume log-normal distribution for the cost. This assumption guarantees that the resulting distributions of WTP are useful and meaningful.

Assuming cost to follow log-normal distribution is not often in non-market valuation, a standard practice is to assume the cost coefficient to be fixed. The three most commonly given reasons for this are: (i) the distribution of the marginal WTP for an attribute is then simply the distribution of that attribute's coefficient; (ii) in this way analysts wish to restrict the price variable to be non-positive for all individuals; (iii) analysts avoid assuming log-normal cost because it is often found to produce behaviorally implausible estimates – i.e. 'exploding' implicit prices (Daly et al. 2012; Carlsson, Frykblom, and Liljenstolpe 2003).

In order to avoid problems with 'exploding' WTP values, in this paper we follow the approach proposed by Giergiczny et al. (2012), i.e. we assumed cost to be log-normally distributed but in order to prevent dividing by very small values we add into the utility function cost/income ratio. This approach allows price sensitivities, hence WTP, to vary with income level. All other non-monetary attributes were assumed to be normally distributed.

References

Carlsson, F., P. Frykblom, and C. Liljenstolpe. 2003. Valuing wetland attributes: an application of choice experiments. *Ecological Economics* 47: 95–103. doi:10.1016/j.ecolecon.2002.09.003.

Daly, A., S. Hess, and K. Train. 2012. Assuring finite moments for willingness to pay in random coefficient models. *Transportation* 39: 19–31. doi:10.1007/s11116-011-9331-3.

Giergiczny, M., S. Valasiuk, M. Czajkowski, M. De Salvo, and G. Signorello. 2012. Including cost income ratio into utility function as a way of dealing with "exploding" implicit prices in mixed logit models. *Journal of Forest Economics* 18: 370–380. doi:10.1016/j.jfe.2012.07.002.

Hensher, D.A., and W.H. Greene. 2003. The Mixed Logit model: The state of practice. *Transportation* 30: 133–176. doi:10.1023/A:1022558715350

McFadden, D. 1974. Conditional logit analysis of qualitative choice behavior. In *Frontiers in Econometrics*, ed. P. Zarembka, 105–142. New York: Academic Press.

Train, K. 2003. *Discrete choice methods with simulation*. Cambridge: Cambridge University Press.
